# Imagination in Autism: A Chance to Improve Early Language Therapy

**DOI:** 10.3390/healthcare9010063

**Published:** 2021-01-11

**Authors:** Andrey Vyshedskiy

**Affiliations:** 1 Biology Department, Boston University, Boston, MA 02215, USA; vysha@bu.edu; Tel.: +1-(617)-433-7724; 2 ImagiRation LLC, Boston, MA 02135, USA

**Keywords:** language therapy, theory of mind, pretend play, symbolic play, language acquisition critical period, critical period, language critical period

## Abstract

Children with autism often have difficulties in imaginative play, Theory of Mind, and playing out different scenarios in their minds. Research shows that the root of these problems may be the voluntary imagination network that involves the lateral prefrontal cortex and its long frontoposterior connections to the temporal-parietal-occipital area. Previously disconnected visuospatial issues (*stimulus overselectivity* and *tunnel vision*) and language issues (lack of comprehension of spatial prepositions and complex recursive sentences) may be explained by the same voluntary imagination deficit. This review highlights the new insights into the mechanism of voluntary imagination, its difference from involuntary imagination, and its unusually strong critical period. Clearer developmental terminology and a better understanding of voluntary imagination have the potential to facilitate communication between therapists and parents, and improve therapy outcomes in children.

## 1. Introduction

**Figure 1:**
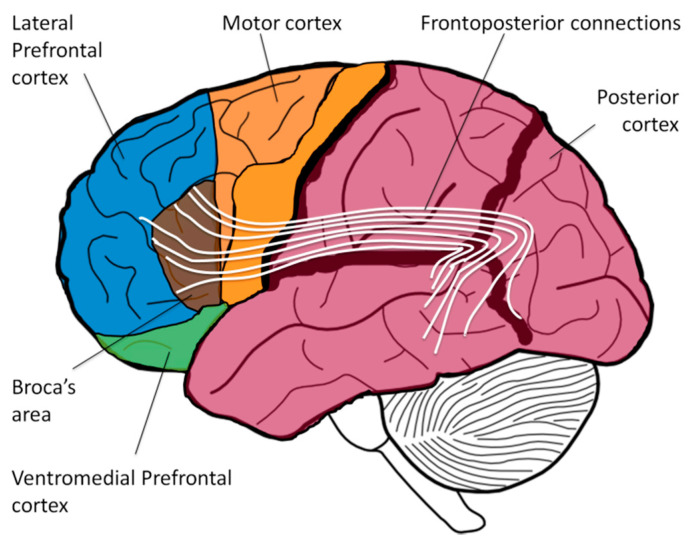
The lateral prefrontal cortex (LPFC) mediates voluntary imagination by exercising control over the posterior cortex via frontoposterior connections, such as arcuate fasciculus and superior longitudinal fasciculus.

Autism is a heterogeneous disorder. Persons on the mild side of the spectrum may face various social challenges but typically have normal voluntary imagination. Conversely, individuals with moderate to severe autism spectrum disorder (ASD) usually manifest a range of deficits related to the voluntary imagination network. Difficulties in the integration of visual features into a global picture are called *stimulus overselectivity*, *tunnel vision*, or *the lack of multi-cue responsivity* [1,2,3,4]; the failure to comprehend spatial prepositions and complex recursive sentences is described as a language deficit; and the inability to interpret others’ beliefs and intentions is referred to as the *Theory of Mind* problem. On the neurological level, though, the origin of these phenomena may stem from a single root: the inefficient frontoposterior connections, such as arcuate fasciculus and superior longitudinal fasciculus (Figure 1). These long fiber tracts connect executive cortical areas in the front (e.g., Broca’s area) with sensory areas in the back (e.g., Wernicke’s area) and have been implicated in various language-related processes in both hemispheres (syntax, semantics, prosody) [5,6,7]. Multiple neurological studies have shown abnormalities in frontoposterior connections of ASD individuals and a significant reduction in the volume of arcuate fasciculus has been hypothesized to be responsible for deficits in Theory of Mind, language, and executive function [8,9].

Complex language comprehension is mediated by the lateral prefrontal cortex (LPFC) exercising its control over the posterior cortex (temporal, parietal, and occipital cortices) via frontoposterior connections. Labeling objects with words is the function of Wernicke’s area [10]; interpreting the grammatical structure of a sentence and assigning word forms to a grammatical group (such as noun, verb, adjective, or preposition) is the function of Broca’s area [10]; but only the LPFC can purposefully juxtapose various mental objects into a novel combination, integrate adjectives and nouns, and organize unrelated visual pieces together [11,12]—all the functions commonly described as *voluntary imagination* [13]. For example, the sentences “The monkey rides the lion” and “The lion rides the monkey” use identical words and the same grammatical structure. Appreciating the delight of the first sentence and the absurdity of the second sentence depends on the LPFC ability to synthesize the mental object of the monkey and the mental object of the lion into a novel scene. (Sentences in which swapping the subject and the object result in a new meaning are called semantically-reversible sentences. By contrast, in a nonreversible sentence, e.g., “The boy writes a letter,” swapping the subject and the object results in a sentence with no real meaning: “A letter writes the boy.”) Similar to semantically-reversible sentences, comprehension of spatial prepositions (e.g., the pen is {*under|in|on|over|beside|behind|in front of*} the table), time prepositions (e.g., touch your nose {*before*|*after*} you touch your ear) and complex recursive sentences (e.g., a snake on the trail, to the left of the tall tree, that is behind the hill) also requires a listener to synthesize several mental objects in front of the mind’s eye using their LPFC [14].

The most complex component of voluntary imagination, Prefrontal Synthesis (PFS), involves the juxtaposition of several mental visuospatial objects into novel combinations [15]. It was hypothesized that the mechanism of PFS involves LPFC-controlled synchronization of *object-encoding neuronal ensembles* (objectNE) in the posterior cortex mediated via the frontoposterior connections (the Neuronal Ensembles Synchronization hypothesis or NES) [6,14,16]. Patients with damage to the LPFC [17], or the frontoposterior fibers [7], or to the temporal-parietal-occipital junction [18] (where objectNEs are encoded) often experience PFS paralysis.

Most lay people assume the presence of innate PFS abilities in all individuals. However, therapists, researchers, and caregivers, working with children exhibiting language comprehension problems, appreciate the challenges of PFS acquisition. Even when language therapy is administered daily, some children never build up their frontoposterior connections essential for PFS. Failure to acquire the PFS ability results in a lifelong inability to understand semantically-reversible sentences, spatial prepositions, recursion, and fairy tales (that require the listener to imagine unrealistic situations). Among individuals diagnosed with Autism Spectrum Disorder (ASD), the prevalence of lifelong PFS paralysis is 30 to 40% [19] and can be as high as 60% among children enrolled in special education schools [20]. These individuals are frequently referred to as having low-functioning ASD. They usually exhibit full-scale IQ below 70 [21,22] and typically perform below the score of 85 in non-verbal IQ tests, see Ref. [22].

What can be done to improve PFS acquisition in children with ASD? In this manuscript, I will argue that wider parent education on several critical issues will result in more effective therapy and better developmental trajectories for children with ASD. These subjects are as follows: (1) the difference between voluntary and involuntary imagination, (2) the strong critical period for PFS acquisition, (3) the dissociation between vocabulary and PFS, and (4) the distinction between a routinized response and PFS ability.

## 2. The Difference between Voluntary and Involuntary Imagination

A vivid and bizarre dream conjures up a myriad of novel mental images. Exactly, the same images can be created volitionally when a person is awake. The neurological mechanisms of these two processes are different [13]. Voluntary combination of mental objects is mediated by the LPFC and patients with damage to the LPFC often lose this ability [17,23,24,25,26,27]. Conversely, the combination of mental objects into novel images during dreaming does not depend on the LPFC: LPFC is inactive during sleep [28,29] and patients whose LPFC is damaged do not notice a change in their dreams [30]. Paradoxically, few scientists are aware of the difference between mechanisms of imagery while dreaming or being awake. Furthermore, neither colloquial English nor scientific jargon have an established way to report on the origin of a conjured up mental image: the term “imagination” is regularly used to describe any experience generated internally whether voluntarily (while being awake) or involuntarily (while being asleep). Only recently have attempts been made to differentiate voluntary and involuntary imagination: Joel Pearson writes in *Nature Reviews*: “When people talk about the mind’s eye, they typically refer to the voluntary experience of creating a conscious sensory experience at will. However, there are many examples of involuntary sensory experiences that are equally decoupled from direct sensory input... One proposed overarching framework is that internal experiences like imagery can be divided into two types of imagery-like experiences, where one is voluntary and the other involuntary” [31].

Involuntary imagination, such as dreaming and insight, is mainly the function of the posterior cortex alone and does not depend on the LPFC or the frontoposterior connections [13]. Voluntary imagination, on the other hand, depends on the LPFC ability to control the posterior cortex via the frontoposterior connections. Establishing, developing and fine-tuning these connections in young children is an experience-dependent process; this experience is primarily driven by the use of recursive language: through normal conversations, story-telling (internal or external), and reading fairy tales. Children who experience fewer conversations show a significant reduction of frontoposterior fiber tracts mediating voluntary imagination [32] and a complete lack of recursive conversations (in feral children and deaf linguistic isolates) is associated with PFS paralysis [11].

In developmental psychology, the problem of PFS paralysis is traditionally described as *stimulus overselectivity, tunnel vision*, or *lack of multi-cue responsivity* [2,3,4]. Affected children have difficulty accomplishing seemingly trivial tasks, such as an instruction to “find a large green pencil” which requires them to combine three different features: the object itself (*pencil*), its size (*large*), and its color (*green*). These children may “overselect” on the word “pencil” and ignore both its size and the fact that it should be also green, therefore, picking up any available pencil; alternatively, they can “overselect” on the color and will pick up any green object; children can also “overselect” on the size and will pick up any large object. (The name of the phenomenon is erroneous. It is not that a child “overselects” on one feature, rather, it is the failure of mental integration. In other words, it is not an attention or focus problem [20], but PFS paralysis).

Children are known to benefit from special exercises that develop voluntary imagination. Speech language pathologists use the techniques of “following directions with increasing complexity”, “combining adjectives, location/orientation, color, and size with nouns”, “building the multiple features/clauses in the sentence” [33] and ABA therapists use “development of multi-cue responsivity” [2], “visual-visual and auditory-visual conditional discrimination” [34,35,36,37], and “reduction of stimulus overselectivity” [3]. However, therapy administration depends on parents recognizing the problem, but they, in turn, often misinterpret any children’s creative constructions as a sign of mature voluntary imagination. In children who are developing atypically, such activities as drawing, Lego constructions, and jigsaw puzzle assembly can be driven exclusively by involuntary imagination and may not reflect the development of the frontoposterior connections and the PFS ability. Vague imagination terminology confuses parents and complicates pediatricians’ work of explaining the problem to the parents. A clear understanding of the difference between voluntary and involuntary imagination as well as unambiguous tests for voluntary imagination acquisition will result in better therapy and improved life outcomes in children with ASD.

## 3. The Strong Critical Period for PFS Acquisition

It is not uncommon for parents to brush off their child’s language delay until the child enters kindergarten at the age of 6, at which time, it may be too late to develop PFS [20,38]. While pediatricians are usually aware of the critical period for language acquisition and normally recommend early intervention at the time of diagnosis, they are often unable to communicate the urgency to parents, due to ambiguity of the critical period concept. Parents are usually familiar with the notion of a critical period, but their familiarity is based on learning a second language in high school. While it is more difficult to learn a second language after puberty [39], it is not impossible: phoneme tuning [40,41], grammar processing [42], articulation control [43], and vocabulary acquisition [44]—all can be significantly improved by training at any age [45,46]. In other words, learning a second language has a weak critical period.

Few parents understand that learning a first language is different from learning a second language, as learning a first language has a strong critical period. Strong critical periods, that make learning impossible after a certain age, are rarely encountered in daily life and, therefore, are counterintuitive. Strong critical periods, however, are common in the central nervous system development. Neural circuits underlying strong critical periods are programmed to be shaped by experience during short periods of early postnatal life; later plasticity is impossible. There are several mechanisms mediating the closure of critical periods. For example, myelination is known to limit the critical period for learning through inhibitory proteins that suppress axon sprouting and synaptogenesis [47]. The most studied examples of strong critical periods include (1) filial imprinting in birds [48], (2) monaural occlusion [49], (3) post-childbirth bonding in mammals [50], (4) the vestibulo-ocular reflex [51], (5) song dialect learning in male white-crowned sparrows [52], and (6) monocular deprivation [53].

Let us look at these abovementioned examples in more detail: (1) Typically, the first moving object seen by baby geese is the mother, and the chick’s survival depends upon learning to follow its mother [54]. Thus, chicks permanently imprint on the first moving object they see shortly after hatching. (2) Sound localization in owls depends on early access to both ears. Plugging one ear (monaural occlusion) in owls during the first two postnatal months results in a lifelong inability to localize sounds [49]. (3) To distinguish their offspring from other lambs in the flock, sheep mothers imprint on their children within a few hours of birth [50]. Later exposure cannot change the initial bonding. (4) The vestibulo-ocular reflex functions to stabilize images on the retinas during head movement. Studies in fish and tadpoles revealed that the vestibulo-ocular reflex can only be acquired during the first 10 days of life; animals who spend the first 10 days in gravity deprivation during a spaceflight end up with a lifelong vestibulo-ocular reflex disability [51]. (5) Marler’s sparrows have song “dialects” acquired in about the first 100 days of life by learning from older males [52]. Once the song is established, further acoustical exposure does not change the song pattern. (6) The most investigated example of the strong critical period has been monocular deprivation [53]. Cats that had one eye sewn shut from birth until three months of age, developed vision only in the open eye. This loss of vision in the covered eye occurs despite there being no damage to the cerebral cortex, the thalamus, or the sensory receptors in the eye. The simple act of closing an eye for the duration of the critical period can profoundly alter the physical structure of the brain and cause a permanent loss of vision through that eye. The duration of this critical period lasts approximately three weeks in mice, three months in cats, and three years in primates [55].

Which component of language has a strong critical period? As mentioned above, vocabulary acquisition [44], articulation control [43], grammar processing [42], and phoneme tuning [40,41]—all can be significantly improved by training at any age [45,46]. PFS, however, seems to have a strong critical period that ends shortly after the age of five. This idea was popularized by Lenneberg and is known as “Lenneberg’s language acquisition critical period hypothesis” [56]. Lenneberg’s conjecture about the strong critical period was based on documented cases of childhood treatment of cancer or epilepsy that resulted in traumatic aphasia and hemispherectomy. When the dominant left hemisphere was surgically removed before the age of five, children often attained normal language and executive functions using the one remaining hemisphere. Conversely, removal of the left hemisphere after the age of five often results in significant impairment of language and executive functions.

Importantly, most pediatricians are uncertain about the distinction between weak and strong critical periods. PFS (formerly called *mental synthesis*) is a relatively new term introduced just two decades ago [57]. Conventionally, PFS was rolled into one of the more general abilities such as language, executive function, cognition, fluid intelligence, Theory of Mind, and working memory. All of these functions have critical periods, but these are weak, as these abilities can be improved well into adulthood [58,59,60]. Indeed, we can improve vocabulary, train self-control, learn new areas of science, improve cognitive speed and working memory at any age. Even Theory of Mind improves at any age when individuals learn mental state vocabulary—particularly linguistic forms for verbs such as ‘think’ and ‘know’ [61]. It is impossible to explain the strong critical period for PFS acquisition to parents without using the narrowly-defined term PFS. Broadly defined terms, such as language, executive function, cognition, fluid intelligence, Theory of Mind, and working memory do not allow pediatricians to clearly explain the urgency of language therapy to parents of newly diagnosed children. Some parents fall back on their intuition of a critical period that is based on learning a second language and conclude that the first language can as well be acquired at a later point. In a number of children, lifelong PFS paralysis is the result of parents’ “wait and see” approach until the child enters kindergarten. The reason for such inaction stems mainly from misunderstanding of the strong critical period for PFS acquisition. The narrowly-defined PFS term will help pediatricians to communicate to parents the concept of a strong critical period and the ensuing urgency of early intensive language therapy.

## 4. Misinterpreting Increasing Vocabulary for Improvement in PFS

Even when intensive language therapy ensues early, success is often hindered by ambiguous goals. As mentioned above, many techniques used by ABA therapists and speech language pathologists are aimed at improving PFS: “development of multi-cue responsivity” [2], “visual-visual and auditory-visual conditional discrimination” [34,35,36,37], “reduction of stimulus overselectivity” [3], “following directions with increasing complexity,” “combining adjectives, location/orientation, color, and size with nouns” and “building the multiple features/clauses in the sentence” [33]. However, PFS exercises are usually just a small part of therapy that mainly focuses on enriching a child’s vocabulary. Vocabulary is easier to train and is highly appreciated by parents. Further encouraging the focus on vocabulary training, the success of an intervention is commonly measured by tests that rely exclusively on a child’s vocabulary, such as the Peabody Picture Vocabulary Test (PPVT-4) [62] and the Expressive Vocabulary Test (EVT-2) [63].

In typical children, PFS acquisition correlates with vocabulary expansion. However, atypically developing children may learn many words without acquiring PFS. In these children, language tests based exclusively on vocabulary assessment may miss the profound deficit in PFS. In order to reproducibly detect the latter, a standardized PFS testing is necessary.

The Preschool Language Scale (PLS-5) is a “go-to” language test for many pediatricians [64]. PLS-5 explores word relationships and concept understanding (self vs. other), but fails to directly assess the PFS ability. The imaginary play section of the PLS-5 primarily depends on involuntary imagination (specifically, categorically-constrained spontaneous imagination [14]) and does not directly assess the PFS ability.

The Token Test for children introduced over five decades ago [65,66], comes close to standardized assessment of the PFS ability. However, it is overly long and complex and, therefore, shunned by psychometricians. There is a clear need for a simple test for PFS.

Recently, our group has developed and validated a 5 min test for PFS called “Language Evaluation of Prefrontal Synthesis” or LEPS [20]. The 10-item LEPS test uses spatial prepositions and non-canonical syntax to present participants with a set of novel questions that children have never encountered before: e.g., (1) Give me a *small red* straw; (2) Put the *blue* cup inside the *red* cup; (3) Inside the *green* cup, put the *orange* cup; (4) Show me: the *monkey* ate the *lion*; (5) Show me: the *giraffe* was eaten by the *elephant*; (6) Put the *lion* on the *giraffe*; (7) Place the *monkey* on top of the *lion* and under the *giraffe*; (8) Imagine an *elephant* and a *chicken*. Which one is bigger? (9) If a *lion* was eaten by a *tiger*, which one is still alive? (10) Imagine the *green* cup inside the *red* cup, which cup is on top?

The sum of 10 items results in the LEPS total score that ranges from 0 (no PFS ability was demonstrated) to 10 (full PFS ability). LEPS test can be used to diagnose PFS paralysis and to monitor PFS acquisition. LEPS norms were calculated in 50 neurotypical children age 2 to 6 years [20]. All children older than 4 years received the LEPS score 7/10 or greater and all children younger than 3 received the LEPS score less than 3/10, indicating that PFS is acquired between the ages of 3 and 4 years.

Among 23 ASD individuals 8 to 21 years (16.4 ± 3.0), 61% received the LEPS score 6/10 or less indicating PFS paralysis [20]. The LEPS score was 90% correct in predicting higher-functioning vs. lower-functioning class assignment in ASD individuals enrolled in a private special education school, compared to only 50% correct by the IQ score. All ASD students with LEPS total score ≥7 were assigned by teachers to higher-functioning classes; all but two students with LEPS total score <7 were assigned by teachers to lower-functioning classes. Thus, the LEPS score 7/10 is the threshold between low-functioning and high-functioning class placement.

LEPS exhibited excellent test–retest reliability, good internal consistency (Cronbach’s alpha = 0.95), good known group validity, and very high inter-observer agreement. LEPS does not rely on productive language and can be used for assessing language in minimally verbal children. The royalty-free LEPS test can be downloaded from [67].

In addition to the LEPS test designed for psychometricians, our group has developed a parent-reported “Mental Synthesis Evaluation Checklist” or MSEC, designed to assess complex language comprehension and PFS acquisition in children [68]. The 20-item MSEC is composed of language comprehension items building steadily in difficulty: e.g., (1) Understands simple stories that are read aloud; (2) Understands elaborate fairy tales that are read aloud (i.e., stories describing fantasy creatures); (6) Understands some simple modifiers (i.e., green apple vs. red apple or big apple vs. small apple); (7) Understands several modifiers in a sentence (i.e., small green apple); (8) Understands size (can select the largest/smallest object out of a collection of objects); (9) Understands possessive pronouns (i.e., your apple vs. her apple); (10) Understands spatial prepositions (i.e., put the apple on top of the box vs. inside the box vs. behind the box); (11) Understands verb tenses (i.e., I will eat an apple vs. I ate an apple); (12) Understands the change in meaning when the order of words is changed (i.e., understands the difference between ‘a cat ate a mouse’ vs. ‘a mouse ate a cat’); (20) Understands explanations about people, objects or situations beyond the immediate surroundings (e.g., “Mom is walking the dog”, “The snow has turned to water”).

For each item, a caregiver is asked how much each of the following is true regarding his/her child on a scale: not true (2 points), somewhat true (1 point), and very true (no points). The sum of 20 items results in the MSEC total score that ranges from 40 (no PFS ability) to 0 (full PFS ability). The MSEC total score 14/40 corresponds to the LEPS score 7/10; the LEPS score >7/10 (higher-functioning class placement), corresponds to the MSEC score <14/40.

The psychometric quality of the MSEC was tested with 3715 ASD children [68]. MSEC exhibited adequate test–retest reliability, good internal reliability (Cronbach’s alpha > 0.9), good known group validity, and good construct validity. The MSEC questionnaire is royalty-free and can be used by parents and researchers to measure receptive language and voluntary imagination [67]. MSEC can be used alone or together with the royalty-free parent-reported “Autism Treatment Evaluation Checklist” (ATEC [69], available at [70]). The ATEC’s four subscales include expressive language, sociability, cognitive awareness and health [69]. The receptive language assessment scale of MSEC provides an important complementary measure of a child’s development.

The LEPS and MSEC evaluations simplify PFS monitoring for both therapists and parents. Regular tracking of PFS over time will aid early identification of children vulnerable to PFS paralysis and help parents, therapists, and pediatricians objectively monitor child improvement once the therapy has been commenced.

## 5. Misinterpreting Routinized Responses for PFS

Another problem inherent to language therapy is routinization. Consider a common exercise that instructs a child to “put the red cup inside the green cup.” There are two ways to successfully complete this stacking cup instruction. One way to find the solution is to mentally synthesize a novel image of the red cup inside the green cup, and then, after completing the mental simulation, arrange the physical objects to match the image in the mind’s eye. An alternative solution could be obtained algorithmically without any imagination: (1) just lift the cup mentioned first and (2) insert it into the cup mentioned second. The latter solution does not require PFS and does not aid the development of frontoposterior connections. It is an automatic action encoded in basal ganglia, akin to other routinized activities, such as tying shoelaces, skiing, riding a bicycle, skating, stopping at a red light, or writing a signature. Training a routinized response does little to improve a child’s general language abilities.

**Figure 2:**
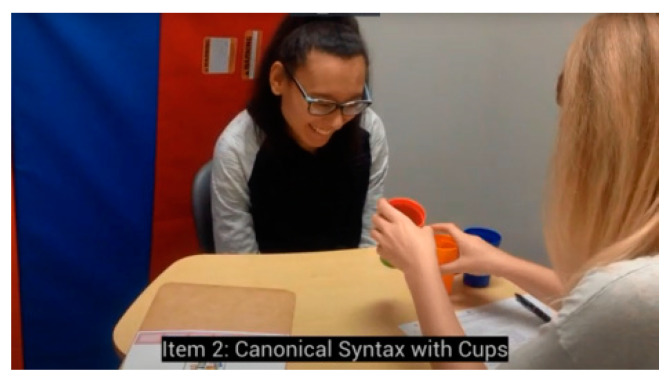
Most ASD (autism spectrum disorder) participants were able to complete the canonical stacking cups task (e.g., “Put the red cup inside the green cup”), but were unable to complete the same task under the condition of non-canonical word order (“Inside the green cup, put the red cup”). Failing ASD participants usually selected the correct cups, but assembled them randomly. https://youtu.be/Hh7pkZB4ETU.

We have recently assessed the problem of routinization in ASD individuals 16 to 20 (17.1 ± 2.0) years of age [20]. Out of 19 participants who were able to complete the canonical stacking cups task (e.g., “Put the red cup inside the green cup”), only 10 (53%) were able to complete the same task under the condition of non-canonical word order (“Inside the green cup, put the red cup”). Contrast this performance with neurotypical children: all but one neurotypical child 3.8 years or older (96%) were able to demonstrate understanding of both canonical and non-canonical instructions. Failing ASD participants usually selected the correct cups, but assembled them randomly (see video recording of a typical ASD student: https://youtu.be/Hh7pkZB4ETU) (Figure 2).

Notably, most failing ASD individuals completed each stacking movement fast, without hesitation. Neurotypical children, on the other hand, normally paused to think before completing the same task, presumably to simulate the answer mentally. The ASD participants in our study have received over 15 years of intensive ABA and language therapy which included the common stacking cups routine. The frequent ‘stacking cups’ training, using canonical syntax only, likely automated students’ responses.

## 6. Conclusions

ASD etiology is highly heterogeneous. Primary neurological problems range from epilepsy [71] to deficient synaptic pruning [72], altered neurotransmitter metabolism [73], disrupted neuronal proliferation [74], mis-migration [74], and synaptic development [74]. These various primary problems result in common secondary problems—social withdrawal and the delay in language acquisition. Social skills and vocabulary, though, can be improved at any age. Conversely, since the use of recursive language in *early* childhood is essential for experience-dependent development of frontoposterior connections [32], language delay can result in the tertiary problem: voluntary imagination deficit and prefrontal synthesis paralysis (PFS paralysis). The tertiary problem of PFS paralysis is a lifelong condition that makes normal conversations, and all, but most primitive explanations impossible due to lack of comprehension of spatial prepositions, semantically-reversible sentences and recursion. Accelerated PFC maturation in ASD individuals [38] can exaggerate this tertiary problem by reducing the duration of the critical period for fine-tuning of frontoposterior connections and PFS acquisition. Language delay combined with the reduced critical period creates a precarious situation where the importance of early intensive language therapy cannot be overstated and where every hour counts.

While DSM-5 lists difficulties in imaginative play as one of the core features of ASD, the attention to imagination has been tangential mostly due to ambiguous terminology. Clearer voluntary imagination terminology and a better understanding of the strong critical period for PFS acquisition have the potential to facilitate communication with parents and focus language therapy on PFS development. Improved PFS tracking with unambiguous tests, such as LEPS and MSEC will allow better monitoring of PFS acquisition by therapists and parents. Synergistic combination of efforts among therapists and parents will result in earlier and more effective therapy, and, eventually, in many more high-functioning productive lives.
